# The Moderating Role of Personality in the Relationship between Internet Use and Study Abroad Difficulties

**DOI:** 10.3390/ijerph18147707

**Published:** 2021-07-20

**Authors:** Gregory-Siy Ching

**Affiliations:** Graduate Institute of Educational Leadership & Development, Research and Development Center for Physical Education Health and Information Technology, Fu Jen Catholic University, New Taipei City 24205, Taiwan; 094478@mail.fju.edu.tw

**Keywords:** adaptability, culture shock, study abroad, higher education, personality

## Abstract

Studying abroad can be stressful due to culture shock and various other difficulties. However, with the current prevalence of information communication technology, we can surmise that study abroad difficulties should be minimal. Since it has been shown that an individual’s personality is highly associated with their internet use behaviors, it would be interesting to determine the effects of personality traits on the relationship between internet use motives and perceived study abroad difficulties. Data were collected from 1870 volunteer study abroad students in Taiwan. Hierarchical regression analysis revealed that when controlling for the effects of age, gender, duration of stay, student status (short-term exchange or degree-seeking), and internet use motives (online benefits, habits, and facilitation), the personality trait neuroticism consistently showed significant relationships with the various study abroad difficulties. Moreover, moderation analyses revealed that all the personality traits except conscientiousness showed significant interactions with internet use, while simple slope comparisons showed significant differences between the high personality traits and their lower counterparts. In sum, an examination of the moderating role of personality traits in the relationship between internet use and study abroad difficulties may be useful for preemptively identifying at-risk students.

## 1. Introduction

The COVID-19 pandemic has brought much uncertainty with respect to studying abroad [1]. The temporary closure of universities and travel bans across the globe have resulted in disruption to study abroad plans. This has resulted in study abroad programs focusing more on shorter travel distances and particularly East Asia as an emerging regional destination hub [2]. For many, study abroad plans have just been delayed or postponed. Recent surveys have indicated that the desire to study abroad is still strong [3,4], prompting universities to reinvent and prepare themselves to facilitate studying abroad in the post-pandemic future [5].

Up until the pandemic, Taiwan was actively involved in promoting international academic exchanges [6]. As a result of the new Southbound Policies [7] and student recruitment in Mainland China [8], in 2019, there were around 130,000 study abroad students in Taiwan [9]. According to the Ministry of Education [10], the majority of the study abroad students in Taiwan are from within the regions of Mainland China, Malaysia, Hong Kong, Japan, Macau, Vietnam, South Korea, Indonesia, Thailand, and India, which are the top ten contributors, making up almost 80% of the international student enrolment. Taiwan is also ranked by Quacquarelli Symonds (QS) as the second best place to study in Asia [11]. In addition, Taiwan also ranked 19th in the QS Higher Education System Strength Rankings with 43 universities entering the latest Asian rankings (five in the top 50 with National Taiwan University in 19th place) [12]. With this having been said, Taiwan study abroad students can be considered as a point of interest.

Studying abroad is not without its challenges [13]. It has been shown to be a stressful undertaking [14,15,16] that can affect an individual’s mental well-being [17,18]. Study abroad students may experience culture shock resulting from their encounters with an unfamiliar culture [19]. Importantly, culture shock is not limited to outgoing students but is also common among returning (or re-entry) students [20,21]. Nonetheless, many still consider that the positive experiences gained from studying abroad are sustainable [22] and outweigh the perceived negative aspects [23]. Hence, it is still considered rewarding for students to participate in study abroad opportunities.

With advances in information communication technology, the internet provides academic sojourners the opportunity for instantaneous communication [24,25] and the facility to document their experiences [26], while also reducing depression levels [27]. Furthermore, studies have found that the internet can provide access to a wide social support network, which can help foster confidence in those studying abroad [28]. At the same time, these familiar co-national social networks provide information and emotional support for individuals in unfamiliar environments [29]. However, too much dependence on these co-national networks may hinder their cultural learning goals [30,31,32]. In general, however, the internet enables students to feel more connected with their home (family and friends), while also facilitating and enhancing their study abroad experiences [33].

Research on studying abroad has found that students’ personality traits are related to their intention and decision to participate in study abroad programs [34,35]. Importantly, intercultural competency development is also related to certain personality traits [36,37]. For instance, intercultural effectiveness is said to be positively correlated with extroversion and openness [38], and conscientious students tend to be more cautious in choosing between study abroad programs [39]. In most of these studies, the five factor model [40] or the big five personality traits [41] are typically used [42]. The personality traits openness, conscientiousness, extraversion, agreeableness, and neuroticism represent the various stable individual differences within the thoughts that people have, the feelings that they experience, and their behaviors [43]. Within study abroad studies, these five personalities are commonly used to understand and describe how students are able to adjust to their new experiences. Research findings have shown that students’ openness is directly associated with their tendency for diversity, which in turn led to better adjustment [31,44], while high levels of agreeableness and openness predict the desire to study abroad [42].

Importantly, personality traits also play an important role as a moderator for technology use [45]. For instance, a study on German and Chinese individuals showed that higher levels of neuroticism and lower levels of conscientiousness are strongly associated with problematic internet use [46]. Furthermore, students’ attitude toward social media was moderated by their degree of openness and neuroticism [47]. Given these findings and those of other studies, which suggest that an individual’s internet use habits are highly related to their personality [48,49,50] and with the environment with which they interact [51], it is therefore interesting to determine whether study abroad difficulties are affected by an individual’s internet use and personality.

Although the relationship between personality traits in study abroad students and internet use has been studied, evidence of the moderator effect of personality traits (openness, conscientiousness, extraversion, agreeableness, and neuroticism) on the relationship between internet use motives and study abroad difficulties is limited. Thus, the objectives of this research are as follows:

- To determine the role of personality traits in predicting study abroad difficulties;
- To determine the moderator effect of personality traits on the relationship between internet use and study abroad difficulties;
- To determine the differences between high and low personality traits with regard to the relationship between internet use and study abroad difficulties.

**Figure 1:**
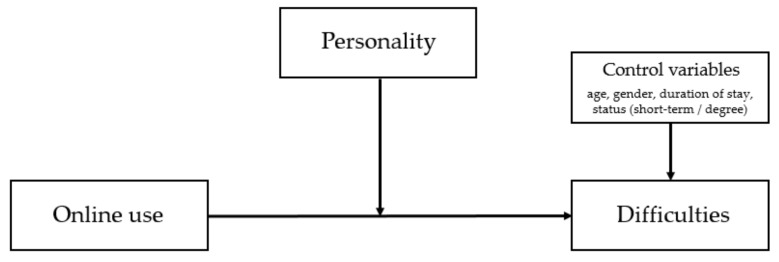
Conceptual diagram of the moderation model.

Age, gender, duration of stay, and status (short-term exchange or degree-seeking) of study abroad students in Taiwan (see Figure 1 for the conceptual diagram of the moderation model) were controlled in the analyses.

## 2. Materials and Methods

### 2.1. Study Design and Participants

The current study is designed as a quantitative study, whereby data are collected using an online survey and later generalized across a group of people to explain a particular phenomenon [52,53]. Posters advertising the study were mailed to international student offices in universities throughout Taiwan. As an incentive, a convenience store cash coupon was offered to the first 500 respondents. A brief description of the study and an explanation of how the collected data would be analyzed and used were provided together with the consent form. Furthermore, participants were informed that the survey included not only personality questions but also their everyday experiences in Taiwan. The study protocol was evaluated and approved by the Fu Jen Catholic University Institutional Review Board.

Data collection and analyses were completed by means of an online survey using the volunteer sampling technique that took place over one 18-week semester during the 2015 academic school year [54]. Sampsize program [55] was used to calculate the minimum sample size. Since there are approximately 112,000 study abroad students in Taiwan during that academic year, a minimum sample size of 383 students was needed for this study (with a 5% margin of error and 95% confidence level). The inclusion criteria included students whose nationality is not Taiwanese and who were enrolled in a university either on a short-term exchange (including Mandarin Chinese language center students) or a degree-seeking program. Foreign students enrolled in senior high schools or lower were excluded in this study.

A total of 1958 volunteer study abroad students in Taiwan participated in the data collection. Of these, 88 students withdrew from the study after reading the informed consent form. Information collected from the remaining 1870 participants were analyzed and screened for outliers, and missing data, which accounted for less than 10% of the entire dataset, were imputed using the expectation maximization algorithm [56,57]. Cronbach’s [58] alpha reliability of the entire survey was computed as 0.84, denoting acceptable internal consistency [59].

Table 1 shows the demographic profile of the students, including the number of female and male participants (female = 925 or 49%, male = 945 or 51%). The average age of participants was around 26 years old. The status, or study abroad type, of the students is also shown in Table 1. Short-term exchange students accounted for 980 (52%) of the participants, while the remaining 890 (48%) were long-term degree-seeking students. Short-term exchange students are typical academic sojourners who are on language programs, cultural immersion stays, and/or academic programs with partner institutions. The typical duration of stay for these exchange students ranges from a few months to a semester and up to a maximum of one year. Degree-seeking students are those formally enrolled in undergraduate or graduate courses with the intention of earning a diploma. The average duration of stay for all the participants was around 15 months.

### 2.2. Measures

For background demographics, participants were asked to provide their age, gender, duration of stay, and status (short-term exchange or degree-seeking). Personality traits were assessed using the 44-item Big Five Inventory (BFI) developed by John and Srivastava [60], which collects self-reported agreement on personal behaviors using a five-point Likert-type [61] scale, with ratings from 1 (least agree) to 5 (most agree). The BFI is a commonly used scale for assessing an individual’s levels of openness, conscientiousness, extraversion, agreeableness, and neuroticism. Openness or openness to experience is a dimension of personality that describes individual differences in seeking, detecting, comprehending, using, and appreciating complex patterns of information, whether sensory or abstract [62]. Conscientiousness can be considered as a tendency to follow socially prescribed norms, to have goals, to plan, and to be self-disciplined [63]. On the other hand, extraversion is characterized by an individual’s ability to successfully engage in various aspects of their lives, and they are generally seen as happy, enthusiastic, confident, energetic, and actively involved throughout their lives [64,65]. Furthermore, agreeableness is an individual difference that refers to the tendency to be likeable, pleasant, and harmonious with others [66]. Finally, neuroticism describes someone who reacts poorly to environmental stress, who interprets ordinary situations as threatening, and who experiences minor frustrations as hopelessly overwhelming [67]. Cronbach’s alpha reliability of the BFI was computed at 0.68, 0.72, 0.68, 0.65, and 0.69, respectively, which indicates that internal consistencies of the BFI were satisfactory.

Internet use motives were assessed using the Study Abroad Internet Use Motives Survey (IUM) developed by Lin and Ching [27] for Taiwan study abroad students, which gathers self-reported agreement on online behaviors using a five-point Likert-type scale, with ratings from 1 (least agree) to 5 (most agree). The IUM is composed of three distinct groups of internet use motives: online benefits, online habits, and online facilitation. Online benefits refer to the notion that the internet is able to alleviate both social and academic difficulties. Online habits or social networking habits refer to how students use social networking sites. Lastly, online facilitation pertains to how students use the internet for social and cultural purposes (p. 1208) [27]. Sample items are “help reduce my academic problems,” “regularly interact with my friends through social media,” and “look for a cultural event that I will attend.” Cronbach’s alpha reliability of the IUM was computed at 0.83, 0.82, and 0.78, respectively, denoting good internal consistencies. For the current sample, confirmatory factor analysis was conducted to verify the factor structure of the observed variables [68,69]. In order to assess the validity of the observed variables, several goodness-of-fit criteria were used. Results show an adequate fit with a chi-squared value of 417.12 at *p* < 0.001 and degrees of freedom (*df*) = 41, root mean square error of approximation (RMSEA) = 0.070 with 90% confidence intervals (CIs) of 0.064 and 0.076, standardized root mean square residual (SRMR) = 0.042, goodness of fit (GFI) = 0.90, Tucker-Lewis index (TLI) = 0.94, and comparative fit index (CFI) = 0.96, all of which are within the recommended ranges [70,71,72,73].

Lastly, study abroad difficulties were examined using the Short-term Study Abroad Situational Change Survey (SSCS) developed by Ching et al. [74] for Taiwan study abroad students, which assesses various self-reported behavioral, cognitive, and affective situational change difficulties using a five-point Likert-type scale with ratings from 1 (least agree) to 5 (most agree). Higher mean scores signify higher study abroad difficulties. The SSCS assesses six distinct groups of study abroad difficulties: academic, leisure living, local viewpoints, daily living, responsive, and suppressive. Academic difficulties consist of the cognitive and behavioral changes which occur within the school environment, while leisure living includes a sense of fun and enjoying oneself, with a focus on getting to know more about Taiwan culture. On the other hand, local viewpoints refer to cognitive interpretations of context that focus on local Taiwanese perspectives. As for daily living, it relates to the changes in general living conditions during study abroad. Responsive difficulties are the students’ need in overcoming difficulties in dealing with odd situations. Lastly, the suppressive factor refers to the usual situations that students are used to in their home country, but which are difficult to replicate in Taiwan (pp. 60–61) [74]. Sample items are “reading and understanding lesson materials,” “going to coffee shops, groceries, or restaurants,” “taking a local perspective on cultural issues,” “adapting to student life in Taiwan,” “dealing with unsatisfactory service,” and “being able to use the things that I’m accustomed to.” Cronbach alpha reliability of the SSCS was computed at 0.86, 0.81, 0.85, 0.81, 0.71, and 0.67, respectively, denoting satisfactory to good internal consistencies. Confirmatory factor analysis was also performed on SSCS, signifying good model fit with a chi-squared value of 1412.30 at *p* < 0.001 and *df* = 194, RMSEA = 0.058 with 90% CIs of 0.055 and 0.061, SRMR = 0.048, GFI = 0.93, TLI = 0.92, and CFI = 0.93.

### 2.3. Statistical Analyses

Data were analyzed using SPSS and AMOS (Version 26.0, IBM Corporation, Armonk, NY, USA) on lease agreement from Hearne Software and the freeware Interaction! Software by Daniel Soper (https://www.danielsoper.com/Interaction/, accessed on 5 January 2021). Confirmatory factor analysis, composite reliability, convergent validity (or the average variance extracted), and discriminant validity to validate the IUM and the SSCS were performed using AMOS. For the confirmatory factor analysis, several criteria were used to evaluate model fit: a significant chi-squared value, RMSEA < 0.08, SRMR < 0.06, and GFI, TLI, and CFI > 0.90 indicate a good fit [70,71,72,73]. Descriptive statistics, such as mean and standard deviation (SD), correlations among the variables, and internal consistencies of BFI, IUM, and SSCS were all computed using SPSS. Independent samples *t*-tests were also performed to determine whether the students’ gender and status (short-term exchange or degree-seeking) had significant effects on their internet use (online benefits, habits, and facilitation), study abroad difficulties (academic, leisure living, local viewpoints, daily living, responsive, and suppressive), and personality (openness, conscientious, extraversion, agreeableness, and neuroticism). Hierarchical multiple regression analyses were then conducted to test for significant relationships between internet use and study abroad difficulties and its subscales while controlling for the background demographic variables age, gender, duration of stay, and status. Lastly, the moderating effect of the different personality traits on the relationship between internet use and study abroad difficulties and a simple slopes comparison between high (+2 SD) and low (−2 SD) personality traits were performed using Interaction! Software [75].

## 3. Results

### 3.1. Descriptive Statistics and Correlations among the Variables

The descriptive results and correlations among the variables are shown in Table 2. The results show that the mean scores of the study abroad difficulties subscales (SSCS factors) ranged from 1.88 to 2.69, signifying moderately low perceived difficulties. The mean scores of the internet use subscales (IUM factors) ranged from 3.51 to 3.81, signifying moderately high perceived agreement. Composite reliability (CR) and convergent validity (or the average variance extracted, AVE) for the SSCS and IUM factors were all above the cutoff points (0.60 for CR and 0.40 for AVE) and are shown in Table 2 [76]. In addition, discriminant validity (DV) was assessed by comparing the square root of AVE with the correlations of the variables. The results show that the DVs were higher than the correlations, signifying adequate construct validity of the SSCS and the IUM [76].

The correlation results show that the study abroad difficulties subscales were positively correlated with each other. Likewise, the internet use subscales were also positively correlated with each other. Interestingly, the study abroad difficulties subscales were mostly negatively correlated with the internet use subscales, implying that as internet use increases, study abroad difficulties decrease. The personality traits openness, conscientiousness, extraversion, and agreeableness were positively correlated with each other but negatively correlated with neuroticism. In addition, openness, conscientiousness, extraversion, and agreeableness were positively correlated with the internet use subscales and negatively correlated with the study abroad difficulties subscales. Neuroticism was negatively correlated with the internet use subscales and positively correlated with the study abroad difficulties subscales, implying that neuroticism is positively linked with study abroad difficulties.

Lastly, duration of stay was negatively correlated with leisure living difficulties, with *r* (1870) = −0.07, *p* < 0.01, and local viewpoints difficulties, with *r* (1870) = −0.05, *p* < 0.05. Likewise, duration of stay was negatively correlated with online benefits, with *r* (1870) = −0.10, *p* < 0.01, and online habits, with *r* (1870) = −0.05, *p* < 0.05. This is interesting because it denotes that students who spent less time studying in Taiwan had higher perceived online benefits and habits. In addition, age was positively correlated with daily living difficulties, with *r* (1870) = 0.11, *p* < 0.01, and suppressive difficulties, with *r* (1870) = 0.11, *p* < 0.01. Surprisingly, age was negatively correlated with all the internet use subscales: online benefits, with *r* (1870) = −0.07, *p* < 0.01; online habits, with *r* (1870) = −0.13, *p* < 0.01; and online facilitation, with *r* (1870) = −0.16, *p* < 0.01. This signifies that older students tend to be less adept at internet usage.

### 3.2. Effects of Gender and Status on Internet Use, Study Abroad Difficulties, and Personality

Independent samples *t*-tests were performed to test whether the students’ gender and status (short-term exchange or degree-seeking) had significant effects on their internet use (online benefits, habits, and facilitation), study abroad difficulties (academic, leisure living, local viewpoints, daily living, responsive, and suppressive), and personality traits (openness, conscientiousness, extraversion, agreeableness, and neuroticism).

The results show that statistically significant differences were found for: suppressive difficulties between females (M = 1.89, SD = 0.73) and males (M = 1.97, SD = 0.84), with *t* (1844) = 2.23, *p* < 0.05; online facilitation between females (M = 3.90, SD = 0.79) and males (M = 3.72, SD = 0.86), with *t* (1860) = 4.83, *p* < 0.001; conscientiousness between females (M = 3.16, SD = 0.62) and males (M = 3.24, SD = 0.62), with *t* (1868) = 2.75, *p* < 0.01; and neuroticism between females (M = 2.87, SD = 0.66) and males (M = 2.80, SD = 0.66), with *t* (1868) = 2.52, *p* < 0.05. Effect sizes were small, ranging from 0.10 to 0.22 [77].

With regard to student status, the results show that statistically significant differences were found for: academic difficulties between short-term exchange (M = 2.19, SD = 0.78) and degree-seeking students (M = 2.29, SD = 0.90), with *t* (1767) = 2.23, *p* < 0.05; daily living difficulties between short-term exchange (M = 2.12, SD = 0.90) and degree-seeking students (M = 2.20, SD = 0.95), with *t* (1825) = 2.01, *p* < 0.05; online facilitation between short-term exchange (M = 3.76, SD = 0.81) and degree-seeking students (M = 3.87, SD = 0.85), with *t* (1868) = 2.85, *p* < 0.01; openness between short-term exchange (M = 3.31, SD = 0.58) and degree-seeking students (M = 3.37, SD = 0.58), with *t* (1868) = 2.14, *p* < 0.05; conscientiousness between short-term exchange (M = 3.15, SD = 0.61) and degree-seeking students (M = 3.25, SD = 0.63), with *t* (1868) = 3.59, *p* < 0.001; agreeableness between short-term exchange (M = 3.52, SD = 0.56) and degree-seeking students (M = 3.63, SD = 0.57), with *t* (1868) = 4.28, *p* < 0.001; and neuroticism between short-term exchange (M = 2.90, SD = 0.65) and degree-seeking students (M = 2.77, SD = 0.66), with *t* (1868) = 4.17, *p* < 0.001. Effect sizes were small, ranging from 0.09 to 0.20.

### 3.3. Variables Associated with Study Abroad Difficulties and Its Subscales

Hierarchical multiple regression analyses were conducted to reveal any significant associations for study abroad difficulties and its subscales: academic, leisure living, local viewpoints, daily living, responsive, and suppressive difficulties. Variables associated with the study abroad difficulties were entered using a three-step procedure. First, to control for possible effects, background demographic variables—age (in years), gender (0 = female, 1 = male), duration of stay (in months), and study abroad status (0 = short-term exchange, 1 = degree-seeking)—were entered into the equation. In the second step, after controlling for the background demographic variables, the various internet use subscales (online benefits, online habits, and online facilitation) were also entered into the equation. Lastly, in the third step, the big five personality traits (openness, conscientiousness, extraversion, agreeableness, and neuroticism) were entered into the equation.

Table 3 shows the results of the hierarchical multiple regression analyses. For study abroad difficulties as a whole, the control variables age (β = 0.098, *t* (1865) = 4.159, *p* < 0.001), duration of stay (β = −0.065, *t* (1865) = −2.528, *p* < 0.05), and status (β = 0.070, *t* (1865) = 2.717, *p* < 0.01) all showed significant associations and together explained 1.20% of the variance (*F* [4, 1865] = 5.797, *p* < 0.001). The internet use subscale online facilitation (β = −0.163, *t* (1862) = −6.135, *p* < 0.001) increases the explained variance to 5% (*F* [3, 1862] = 25.096, *p* < 0.001). Finally, agreeableness (β = −0.127, *t* (1857) = −5.037, *p* < 0.001) and neuroticism (β = 0.167, *t* (1857) = 6.181, *p* < 0.001) increased the explained variance to 12.30% (*F* [5, 1857] = 30.894, *p* < 0.001).

For the study abroad difficulties subscale academic difficulties, the only control variable that revealed a significant association was student status (β = 0.066, *t* (1865) = 2.562, *p* < 0.01), which explained 0.50% of the variance (*F* [4, 1865] = 2.546, *p* < 0.05). Next, the internet use subscale online facilitation (β = −0.170, *t* (1862) = −6.405, *p* < 0.001) increased the explained variance to 4.30% (*F* [3, 1862] = 24.670, *p* < 0.001). Then, conscientiousness (β = −0.115, *t* (1857) = −4.445, *p* < 0.001) and neuroticism (β = 0.111, *t* (1857) = 4.057, *p* < 0.001) increased the explained variance to 10.30% (*F* [5, 1857] = 24.683, *p* < 0.001).

For the study abroad difficulties subscale leisure living difficulties, the control variables age (β = 0.052, *t* (1865) = 2.177, *p* < 0.05) and duration of stay (β = −0.092, *t* (1865) = −3.575, *p* < 0.001) revealed significant associations and explained 0.90% of the variance (*F* [4, 1865] = 4.329, *p* < 0.01). Next, the internet use subscale online facilitation (β = −0.177, *t* (1862) = −6.680, *p* < 0.001) increased the explained variance to 4.80% (*F* [3, 1862] = 25.168, *p* < 0.001). Finally, agreeableness (β = −0.096, *t* (1857) = −3.702, *p* < 0.001) and neuroticism (β = 0.135, *t* (1857) = 4.891, *p* < 0.001) increased the explained variance to 8.40% (*F* [5, 1857] = 14.785, *p* < 0.001).

For the study abroad difficulties subscale local viewpoints, the control variables age (β = 0.054, *t* (1865) = 2.267, *p* < 0.05) and duration of stay (β = −0.073, *t* (1865) = −2.824, *p* < 0.01) revealed significant associations and explained 0.70% of the variance (*F* [4, 1865] = 3.490, *p* < 0.01). The internet use subscale online benefits (β = −0.108, *t* (1862) = −3.928, *p* < 0.001) increased the explained variance to 2.20% (*F* [3, 1862] = 9.703, *p* < 0.001), and conscientiousness (β = −0.073, *t* (1857) = −2.752, *p* < 0.01) and neuroticism (β = 0.117, *t* (1857) = 4.163, *p* < 0.001) increased the explained variance to 5.30% (*F* [5, 1857] = 12.264, *p* < 0.001).

For the study abroad difficulties subscale daily living difficulties, the control variables age (β = 0.123, *t* (1865) = 5.201, *p* < 0.001), duration of stay (β = −0.051, *t* (1865) = −1.979, *p* < 0.05), and status (β = 0.078, *t* (1865) = 3.041, *p* < 0.01) all revealed significant associations and explained 1.70% of the variance (*F* [4, 1865] = 8.104, *p* < 0.001). The internet use subscale online facilitation (β = −0.094, *t* (1862) = −3.485, *p* < 0.001) increased the explained variance to 2.70% (*F* [3, 1862] = 6.071, *p* < 0.001), and conscientiousness (β = 0.083, *t* (1857) = 3.193, *p* < 0.001), agreeableness (β = −0.202, *t* (1857) = −7.848, *p* < 0.001), and neuroticism (β = 0.123, *t* (1857) = 4.452, *p* < 0.001) further increased the explained variance to 8.80% (*F* [5, 1857] = 24.903, *p* < 0.001).

For the study abroad difficulties subscale responsive difficulties, none of the background demographics showed significant associations. The internet use subscales online habits (β = −0.056, *t* (1862) = −1.984, *p* < 0.05) and online facilitation (β = −0.098, *t* (1862) = −3.652, *p* < 0.001) explained 1.70% of the variance (*F* [3, 1862] = 9.475, *p* < 0.001). Then, extraversion (β = −0.084, *t* (1857) = −3.248, *p* < 0.001), agreeableness (β = −0.114, *t* (1857) = −4.391, *p* < 0.001), and neuroticism (β = 0.132, *t* (1857) = 4.740, *p* < 0.001) increased the explained variance to 7.40% (*F* [5, 1857] = 22.949, *p* < 0.001).

Lastly, for the study abroad difficulties subscale suppressive difficulties, the only control variable with a significant association was age (β = 0.111, *t* (1865) = 4.681, *p* < 0.001), which explained 1.40% of the variance (*F* [4, 1865] = 6.797, *p* < 0.001). The internet use subscales online habits (β = −0.055, *t* (1862) = −2.001, *p* < 0.05) and online facilitation (β = −0.136, *t* (1862) = −5.109, *p* < 0.001) increased the explained variance to 3.80% (*F* [3, 1862] = 15.231, *p* < 0.001), and agreeableness (β = −0.097, *t* (1857) = −3.697, *p* < 0.001) and neuroticism (β = 0.086, *t* (1857) = 3.056, *p* < 0.01) further increased the explained variance to 5.70% (*F* [5, 1857] = 7.402, *p* < 0.001).

### 3.4. Testing the Moderating Effect of Personality Traits

To understand the moderating effect of the different personality traits, several moderation analyses were performed using Interaction! Software [75]. In addition to the moderation analyses, simple slopes difference tests were used to determine the three-way interactions within the moderated multiple regression models [78]. More specifically, the simple slopes difference tests were used to test the effects of extreme values [79]—high (+2 SD) personality traits and their lower (−2 SD) counterparts—on the relationship between internet use and study abroad difficulties. For better interpretability of the results, all variables and predictors were standardized and centered prior to computing [80].

**Figure 2:**
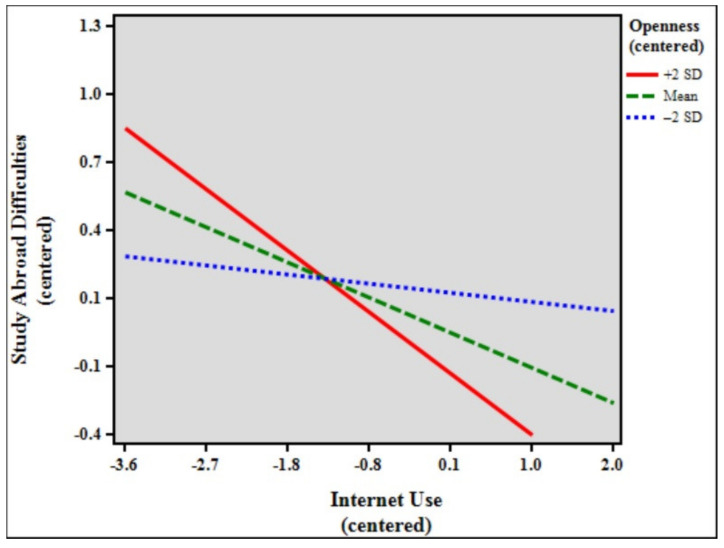
Simple slope plot for the moderation effect of openness.

Table 4 shows the results of the moderation analysis and simple slopes models of study abroad difficulties, internet use, and openness. The total model accounted for 5.30% (*F* [7, 1862] = 14.913, *p* < 0.001) of the variance in study abroad difficulties. The results indicate that the control variables age (β = 0.074, *p* < 0.01), duration of stay (β = −0.074, *p* < 0.01), and status (β = 0.081, *p* < 0.01) significantly predicted study abroad difficulties. In addition, internet use (β = −0.156, *p* < 0.001), openness (β = −0.078, *p* < 0.01), and the interaction between internet use and openness (β = −0.058, *p* < 0.01) were statistically significant in the model. The effect size of the interaction was very small, with f^2^ = 0.06 [81]. Simple slopes difference analysis showed that the relationship between internet use and study abroad difficulties was significant among high (slope β = −0.272, *p* < 0.001) and low (slope β = −0.040, *p* > 0.05, non-significant or ns) openness students (β = −0.231, *p* < 0.001) [82]. Figure 2 shows the simple slope plot for the moderation effect of openness, which signifies that openness strengthens the negative relationship between internet use and study abroad difficulties.

**Figure 3:**
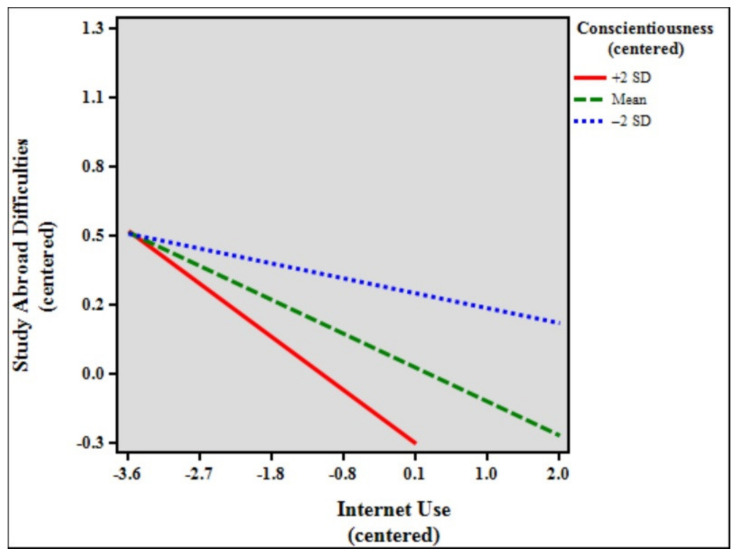
Simple slope plot for the moderation effect of conscientiousness.

Table 5 shows the results of the moderation analysis and simple slopes models of study abroad difficulties, internet use, and conscientiousness. The total model accounted for 6.51% (*F* [7, 1862] = 18.511, *p* < 0.001) of the variance in study abroad difficulties. The results indicate that the control variables age (β = 0.067, *p* < 0.01), duration of stay (β = −0.075, *p* < 0.01), and status (β = 0.088, *p* < 0.001) significantly predicted study abroad difficulties. In addition, internet use (β = −0.143, *p* < 0.001) and conscientiousness (β = −0.143, *p* < 0.001) were statistically significant, although the interaction between internet use and conscientiousness (β = −0.040, *p* > 0.05, ns) was not statistically significant in the model. Simple slopes difference analysis showed that the relationship between internet use and study abroad difficulties was significant among high (slope β = −0.223, *p* < 0.001) and low (slope β = −0.062, *p* > 0.05, ns) conscientiousness students (β = −0.161, *p* < 0.001). Figure 3 shows the simple slope plot for the moderation effect of conscientiousness, signifying that conscientiousness strengthens the negative relationship between internet use and study abroad difficulties.

**Figure 4:**
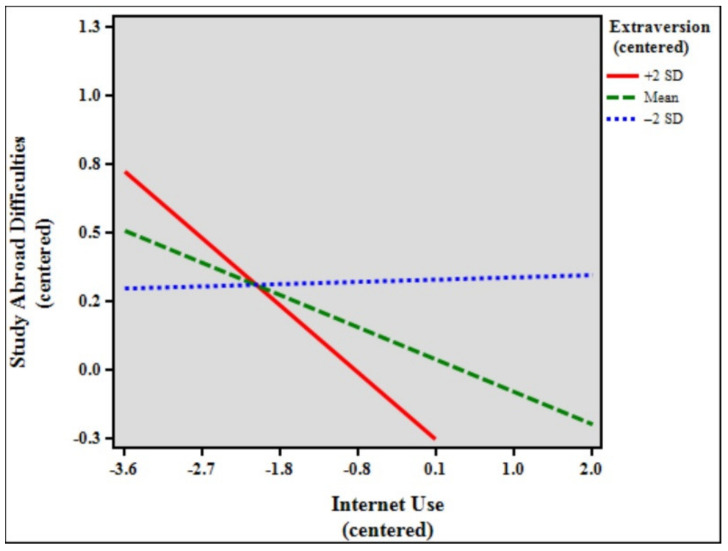
Simple slope plot for the moderation effect of extraversion.

Table 6 shows the results of the moderation analysis and simple slopes models of study abroad difficulties, internet use, and extraversion. The total model accounted for 7.15% (*F* [7, 1862] = 20.471, *p* < 0.001) of the variance in study abroad difficulties. The results indicate that the control variables age (β = 0.072, *p* < 0.01), duration of stay (β = −0.073, *p* < 0.01), and status (β = 0.075, *p* < 0.01) significantly predicted study abroad difficulties. In addition, internet use (β = −0.137, *p* < 0.001), extraversion (β = −0.151, *p* < 0.001), and the interaction between internet use and extraversion (β = −0.073, *p* < 0.001) were statistically significant in the model. The effect size of the interaction was very small, with f^2^ = 0.08. Simple slopes difference analysis showed that the relationship between internet use and study abroad difficulties was significant among high (slope β = −0.283, *p* < 0.001) and low (slope β = 0.010, *p* > 0.05, ns) extraversion students (β = −0.293, *p* < 0.001). Figure 4 shows the simple slope plot for the moderation effect of extraversion, signifying that extraversion strengthens the negative relationship between internet use and study abroad difficulties.

**Figure 5:**
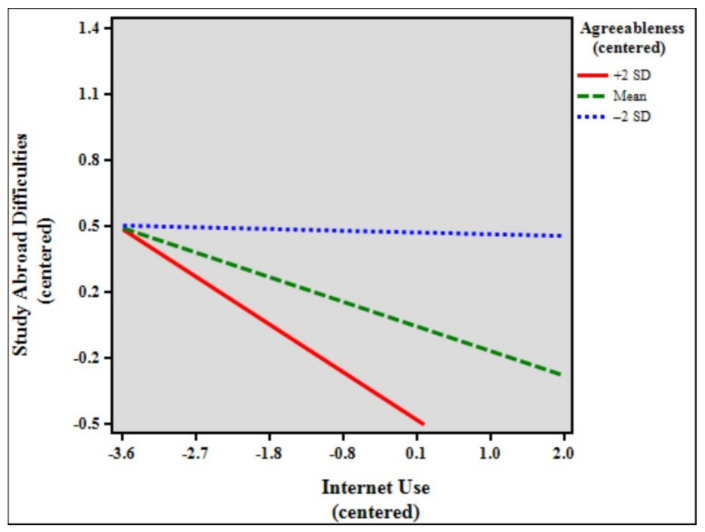
Simple slope plot for the moderation effect of agreeableness.

Table 7 shows the results of the moderation analysis and simple slopes models of study abroad difficulties, internet use, and agreeableness. The total model accounted for 9.06% (*F* [7, 1862] = 26.512, *p* < 0.001) of the variance in study abroad difficulties. The results indicate that the control variables age (β = 0.068, *p* < 0.01), duration of stay (β = −0.074, *p* < 0.01), and status (β = 0.094, *p* < 0.001) significantly predicted study abroad difficulties. In addition, internet use (β = −0.125, *p* < 0.001), agreeableness (β = −0.216, *p* < 0.001), and the interaction between internet use and agreeableness (β = −0.058, *p* < 0.01) were statistically significant in the model. The effect size of the interaction was very small, with f^2^ = 0.10. Simple slopes difference analysis showed that the relationship between internet use and study abroad difficulties was significant among high (slope β = −0.242, *p* < 0.001) and low (slope β = −0.008, *p* > 0.05, ns) agreeableness students (β = −0.234, *p* < 0.001). Figure 5 shows the simple slope plot for the moderation effect of agreeableness, signifying that agreeableness strengthens the negative relationship between internet use and study abroad difficulties.

**Figure 6:**
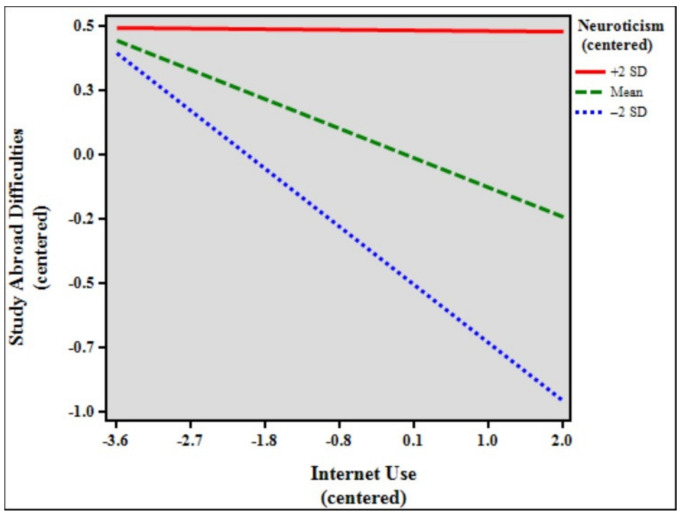
Simple slope plot for the moderation effect of neuroticism.

Table 8 shows the results of the moderation analysis and simple slopes models of study abroad difficulties, internet use, and neuroticism. The total model accounted for 10.60% (*F* [7, 1862] = 31.551, *p* < 0.001) of the variance in study abroad difficulties. The results indicate that the control variables age (β = 0.072, *p* < 0.01), duration of stay (β = −0.069, *p* < 0.01), and status (β = 0.097, *p* < 0.001) significantly predicted study abroad difficulties. In addition, internet use (β = −0.123, *p* < 0.001), neuroticism (β = 0.242, *p* < 0.001), and the interaction between internet use and neuroticism (β = 0.060, *p* < 0.01) were statistically significant in the model. The effect size of the interaction was very small, with f^2^ = 0.12. Simple slopes difference analysis showed that the relationship between internet use and study abroad difficulties was significant among high (slope β = −0.003, *p* > 0.05, ns) and low (slope β = −0.242, *p* < 0.001) neuroticism students (β = 0.239, *p* < 0.001). Figure 6 shows the simple slope plot for the moderation effect of neuroticism, signifying that neuroticism dampens the negative relationship between internet use and study abroad difficulties.

## 4. Discussion

The primary objective of the current study was to examine the moderating effects of personality traits on the relationship between internet use and study abroad difficulties. To achieve this, several analyses were performed. The descriptive statistics show that local viewpoints (M = 2.69, SD = 0.96) ranked highest among the difficulties faced by study abroad students. Local viewpoints are issues related to understanding the Taiwanese world view and local perspectives on cultural issues and seeing things from a Taiwanese perspective [74]. Gaining insights into the local perspective is an important component of cross-cultural understanding and awareness [83]. Although participating in study abroad programs can help develop an individual’s cross-cultural awareness [84], the key to building cross-cultural understanding is still actual physical interaction with host country nationals [85,86]. Local immersion can often be difficult, but cultural understanding is needed to gain insight into local values [87]. Study abroad students must have a desire to interact with host nationals (in the current case, Taiwanese people) [88], as without this, cross-cultural understanding and awareness will be difficult to achieve.

The results also show that leisure living (M = 1.88, SD = 0.80) ranks lowest among the difficulties. Leisure living refers to issues pertaining to the cultural tourism aspect of studying abroad. Low perceived difficulties are noted on issues that relate to sightseeing, shopping, buying groceries, eating in restaurants, and using the mass transportation system [74]. The concept of educational or cultural tourism is an approach in study abroad that serves as a starting point for more in-depth and meaningful interaction with host nationals [89,90]. The current results echo previous findings, which have highlighted the importance of local tourism as one of the major pull factors for study abroad students in Taiwan [91].

Regarding the study abroad students’ internet use motives, online facilitation (M = 3.81, SD = 0.83) ranked highest. Online facilitation refers to issues relating to students’ use of the internet to facilitate social and cultural activities [27]. This is related to the area of leisure living, as students tend to use the internet to find information on places to visit, cultural events to attend, and other leisure activities. More importantly, all the internet use subscales were significantly and negatively correlated with all the study abroad difficulties subscales (except for the correlation between daily living and online benefits, which is not significant), signifying that internet use somehow minimizes study abroad difficulties, as noted in previous studies [27,92]. For instance, in the current study, online benefits are regarded as factors that can help reduce both social and academic difficulties, while online habits—or, more specifically, social media habits—help students stay connected with their family and friends. In essence, the findings suggest that internet use has positive effects in reducing study abroad difficulties.

The personality traits of students were also collected, with agreeableness (M = 3.57, SD = 0.56) ranking as the most evident, openness (M = 3.34, SD = 0.58) coming second, and neuroticism (M = 2.84, SD = 0.66) last. This finding supports the results of a previous study conducted in Germany, which found that students who participated in study abroad programs scored higher for agreeableness and openness [42]. Interestingly, correlational analyses showed that neuroticism was significantly and negatively correlated with the other personality traits and with all the internet use subscales. Importantly, neuroticism is significantly and positively correlated with all the study abroad difficulties subscales. This implies that students who rate high for neuroticism tend to have more study abroad difficulties and less internet use. This finding aligns with Kong’s [93] suggestion that neuroticism is related to study abroad anxiety and affects an individual’s sociocultural adaptation [94].

As for the duration of stay, the findings show negative correlations with leisure living and local viewpoints difficulties, as well as with online benefits and online habits. These are expected and signify that the longer a student stays in Taiwan, the more acquainted they become with host nationals and, hence, the fewer local viewpoints difficulties they experience. Researchers have pointed out that longer duration of stay increases the chances of interaction with host nationals [95,96] and, hence, contributes to a better understanding of the local culture and values. At the same time, the longer a student remains in Taiwan, the more opportunities they have to travel around the country. Furthermore, students who spent less time in Taiwan had significantly higher perceived online benefits and habits. The findings are also not surprising, as they suggest that students who are new to studying abroad (those who have spent less time in Taiwan) tend to be more engaged in their academic work and are more likely to be more connected with their family and friends.

The findings also show that younger students tend to have difficulties with daily living and with suppressing their emotions. More specifically, younger students seem to have greater difficulty adjusting to the local etiquette and student life in Taiwan. In addition, they might be unable to cope with the many affective changes that are linked to living and studying in a foreign country. This aligns somewhat with a study on Korean study abroad students, which found that younger students were more reluctant to seek help from others with their adjustment difficulties [97]. Lastly, the findings show that older students tend to be less adept at using the internet. Although it has been suggested that older individuals tend to refrain from technology use, however, this typically applies to those over 65 years of age [98]. In fact, some researchers have proposed that the perceived usefulness and intention regarding internet use do not change with age [99].

After the descriptive analyses were completed, the various variables were assessed for gender differences. An independent samples *t*-test showed that male students had greater difficulty in suppressing their emotions than their female counterparts. This finding is rather unique, as most previous studies have found that male students are more emotionally stable than female students [36,100]. Gender differences were also found for neuroticism, with female students rating slightly higher than male students. Since neuroticism is related to emotional stability [101], so it follows that female students would be more emotionally unstable than men. Gender differences were also found in online facilitation, whereby female students were more likely to use the internet to facilitate their social and cultural activities than male students. In addition, male students were also found to be more conscientious than female students.

Additional independent samples *t*-tests were performed on study abroad students’ status. The findings show that degree-seeking students rated significantly higher for academic and daily living difficulties. Academic difficulties are issues related to the teaching and learning processes during lectures and fulfilling school work. This finding coincides with those of numerous previous researchers, who have reported that academic stress and pressure to succeed are experienced by Asian students studying in the United States and Western students studying in China [29,102,103]. Similarly, significant differences were found regarding online facilitation and the personality traits openness, conscientiousness, and agreeableness, with degree-seeking students rating higher than short-term exchange students. By contrast, short-term exchange students rated significantly higher for neuroticism than degree-seeking students.

To determine the role of personality traits in predicting study abroad difficulties, several hierarchical multiple regression analyses were conducted. The background demographic variables age, gender, duration of stay, and status were used as control variables in the analyses (stage 1). Similarly, internet use subscales (online benefits, online habits, and online facilitation) were held constant (stage 2). An overall summary of the hierarchical multiple regression analyses is shown in Table 9. The findings show that when controlling for the background demographics and internet use subscales, the personality trait neuroticism consistently showed a positive association with study abroad difficulties. By contrast, some of the other personality traits (except openness) were found to have significant negative effects on study abroad difficulties, although these results were not as consistent.

Widiger [67] explained neuroticism as the tendency of individuals to experience negative emotions. He also reported that individuals who rated high for neuroticism are more likely to experience anxiety and depression. In study abroad students, neuroticism is commonly correlated with the stress associated with experiencing something new and unfamiliar [93,94]. In a longitudinal study, Jeronimus et al. [104] found that neuroticism consistently predicted negative experiences. However, both Andrews et al. [105] and Niehoff et al. [42] suggested that study abroad experiences help reduce the levels of neuroticism. The findings of the current study suggest that neuroticism is closely related to study abroad difficulties. In other words, students who rated high for neuroticism tended to experience greater difficulty while studying abroad.

When examining the moderating effects of personality traits on the relationship between internet use and study abroad difficulties, background demographics were treated as control variables. The findings reveal that openness, extraversion, agreeableness, and neuroticism all showed significant interactions with internet use, suggesting a moderating effect on the relationship between internet use and study abroad difficulties. In other words, although neuroticism by itself is positively associated with study abroad difficulties, when the relationship between internet use and study abroad difficulties was considered, openness, extraversion, agreeableness, and neuroticism all exhibited a moderating role. More specifically, openness, extraversion, and agreeableness were found to strengthen the negative relationship between internet use and study abroad difficulties, while neuroticism was found to strengthen the positive relationship between internet use and study abroad difficulties. These findings are unique and contribute to a better understanding of how individual personality traits affect study abroad experiences.

Lastly, to further understand the effects of extreme personalities—high personality traits (+2 SD) and low personality traits (−2 SD)—simple slopes difference tests were performed. The findings show significant differences between all the high personality traits and their lower counterparts. Further analyses of the results revealed particularly interesting findings. While the moderating effect of conscientiousness was not statistically significant in the previous analysis, simple slopes comparisons showed that when the sample was separated into high (+2 SD, *n* = 48), mean (*n* = 1781), and low (−2 SD, *n* = 41) conscientiousness, the moderating effects of both high and mean conscientiousness were, in fact, significant (see Table 5, simple slopes models). Furthermore, for the personality traits openness, extraversion, and agreeableness, the moderating effects of low (−2 SD) ratings were also not significant. Figure 2, Figure 4, and Figure 5 show that low (blue broken lines) ratings for personality traits exhibit small to very small slopes (almost a straight line for agreeableness), signifying that study abroad difficulties are almost not affected at all by internet use. Likewise, the moderating effect of high (+2 SD) neuroticism was not significant. Importantly, Figure 6 shows that the slope for high neuroticism (red line) is almost a straight line, denoting that study abroad difficulties are not affected at all by internet use.

It should be noted that this study is not without limitations. The data analysis excludes some information regarding students’ personal, situational, and contextual characteristics that may also influence the difficulties they may face when studying abroad, which is currently beyond the scope of the study. For instance, these include students’ country of origin, Mandarin Chinese language proficiency, discipline of study, housing, host institutions’ ranking, governance, and location. Future studies are encouraged to examine these variables either as a predictor or as an antecedent of study abroad difficulties.

## 5. Conclusions

In sum, this study reveals several pertinent findings. First, descriptive, correlational, and group (independent samples *t*-tests) analyses showed that background demographic variables seemed to exert some influence on internet use, study abroad difficulties, and personality traits. Second, background demographic variables and internet use were controlled to determine for the effects of personality traits on study abroad difficulties. The findings show that neuroticism consistently exhibited positive associations with study abroad difficulties. Third, to determine the moderating effects of personality traits on the relationship between internet use and study abroad difficulties, background demographic variables were controlled. The findings reveal that all personality traits except conscientiousness (openness, extraversion, agreeableness, and neuroticism) showed significant interactions with internet use, which implies that these personality traits do moderate the relationship between internet use and study abroad difficulties. Lastly, to further understand the effects of extreme personalities, a comparison between high (+2 SD) and low (−2 SD) personality traits was performed using simple slope differences while controlling for the background demographic variables. All variables were standardized and centered prior to the analyses. The findings show significant differences between all the high personality traits and their lower counterparts, which suggests that while some personality traits moderate the relationship between internet use and study abroad difficulties, the levels of the personality traits also matter.

Apart from the fact that international student offices can use personality scales to identify at-risk students, some practical implications can also be drawn. For instance, international student offices can organize self-discovery workshops, so students can also be made aware of their personalities. Furthermore, interaction between the local Taiwanese students can be encouraged with the help of study groups for academic assistance and sightseeing tours for tourism purposes. In addition, with the help of international student offices, study abroad students can also act as cultural ambassadors and help promote their home country. Ultimately, increased self-awareness, self-understanding, and interaction with the local community should help ease the students’ acculturation process and therefore lead to a more satisfying study abroad experience.

## Figures and Tables

**Table 1:** Demographic profile of the participants.

Demographics	Classification	*n*	*%*
Gender	Female	925	49
	Male	945	51
	Total	1870	100
Status	Short-term exchange	980	52
	Degree-seeking	890	48
	Total	1870	100

Note. *n* = 1870.

**Table 2:** Descriptive statistics, discriminant validity, and correlation matrix of the variables.

Variables	PS	Mean	SD	CR	AVE	DV ^1^	1	2	3	4	5	6	7	8	9	10	11	12	13	14	15	16
1. Academic	1~5	2.24	0.84	0.86	0.51	0.71	**0.86**	0.48 **	0.40 **	0.39 **	0.44 **	0.40 **	−0.11 **	−0.12 **	−0.19 **	−0.14 **	−0.22 **	−0.20 **	−0.16 **	0.23 **	0.03	0.04
2. Leisure living	1~5	1.88	0.80	0.82	0.53	0.73		**0.81**	0.33 **	0.40 **	0.40 **	0.51 **	−0.08 **	−0.12 **	−0.20 **	−0.11 **	−0.11 **	−0.13 **	−0.19 **	0.21 **	−0.07 **	0.04
3. Local viewpoints	1~5	2.69	0.96	0.85	0.66	0.82			**0.85**	0.31 **	0.35 **	0.25 **	−0.12 **	−0.07 **	−0.08 **	−0.09 **	−0.15 **	−0.11 **	−0.09 **	0.17 **	−0.05 *	0.04
4. Daily living	1~5	2.16	0.92	0.82	0.61	0.78				**0.81**	0.45 **	0.50 **	−0.05	−0.06 **	−0.11 **	−0.04	−0.05 *	−0.11 **	−0.23 **	0.18 **	−0.00	0.11 **
5. Responsive	1~5	2.47	0.92	0.72	0.46	0.68					**0.71**	0.37 **	−0.05 *	−0.09 **	−0.11 **	−0.07 **	−0.13 **	−0.18 **	−0.20 **	0.22 **	−0.02	0.03
6. Suppressive	1~5	1.93	0.79	0.68	0.42	0.65						**0.67**	−0.06 **	−0.11 **	−0.17 **	−0.06 **	−0.08 **	−0.07 **	−0.16 **	0.14 **	0.01	0.11 **
7. Online benefits	1~5	3.52	0.93	0.84	0.56	0.75							**0.83**	0.50 **	0.42 **	0.13 **	0.11 **	0.10 **	0.08 **	−0.07 **	−0.10 **	−0.07 **
8. Online habits	1~5	3.51	0.94	0.82	0.54	0.73								**0.82**	0.45 **	0.21 **	0.20 **	0.23 **	0.24 **	−0.19 **	−0.05 *	−0.13 **
9. Online facilitation	1~5	3.81	0.83	0.79	0.57	0.75									**0.78**	0.22 **	0.15 **	0.23 **	0.24 **	−0.20 **	0.02	−0.16 **
10. Openness	1.5~5	3.34	0.58													**0.68**	0.29 **	0.28 **	0.24 **	−0.21 **	0.03	−0.01
11. Conscientiousness	1.33~5	3.20	0.62														**0.72**	0.31 **	0.35 **	−0.45 **	0.01	−0.07 **
12. Extraversion	1.25~5	3.21	0.62															**0.68**	0.27 **	−0.42 **	−0.01	−0.02
13. Agreeableness	1.56~5	3.57	0.56																**0.65**	−0.43 **	0.03	−0.06 **
14. Neuroticism	1~5	2.84	0.66																	**0.69**	−0.04	0.03
15. Duration	1~312	15	23																			0.12 **
16. Age	17~57	26	7																			

Notes. *n* = 1870, PS = possible scores, SD = standard deviation, CR = composite reliability, AVE = average variance extracted, and DV = discriminant validity. ^1^ Square root of AVE. Numbers 1 to 16 correspond to the variables. Duration is in months. Age is in years. * *p* < 0.05, ** *p* < 0.01. Internal consistency values: Cronbach’s alpha coefficients are on diagonals (values in bold). Pearson correlation coefficients are above the diagonals.

**Table 3:** Hierarchical multiple regression analyses of study abroad difficulties.

	Predictors	*F* Change	*t*	df	B	SE	β	R^2^ Change
A. Dependent variable: Total study abroad difficulties								
I.	Constant				1.986	0.059		
	Control variables	5.797 ***		4, 1865				0.012
	Age		4.159 ***		0.009	0.002	0.098	
	Gender		−0.120		−0.003	0.029	−0.003	
	Duration of stay		−2.528 *		−0.002	0.001	−0.065	
	Status		2.717 **		0.086	0.032	0.070	
II.	Internet use	25.096 ***		3, 1862				0.038
	Online benefits		−0.585		−0.011	0.018	−0.016	
	Online habits		−1.883		−0.034	0.018	−0.052	
	Online facilitation		−6.135 ***		−0.120	0.020	−0.163	
III.	Personality	30.894 ***		5, 1857				0.073
	Openness		−0.437		−0.011	0.025	−0.010	
	Conscientiousness		−0.857		−0.022	0.025	−0.022	
	Extraversion		−1.869		−0.046	0.025	−0.047	
	Agreeableness		−5.037 ***		−0.138	0.027	−0.127	
	Neuroticism		6.181 ***		0.156	0.025	0.167	
B. Dependent variable: Academic difficulties								
I.	Constant				2.049	0.080		
	Control variables	2.546 *		4, 1865				0.005
	Age		1.878		0.006	0.003	0.045	
	Gender		0.022		0.001	0.039	0.001	
	Duration of stay		−0.332		0.000	0.001	−0.009	
	Status		2.562 **		0.111	0.043	0.066	
II.	Internet use	24.670 ***		3, 1862				0.038
	Online benefits		−0.421		−0.010	0.025	−0.011	
	Online habits		−1.533		−0.038	0.025	−0.042	
	Online facilitation		−6.405 ***		−0.171	0.027	−0.170	
III.	Personality	24.683 ***		5, 1857				0.060
	Openness		−1.445		−0.050	0.034	−0.035	
	Conscientiousness		−4.445 ***		−0.155	0.035	−0.115	
	Extraversion		−2.725 **		−0.093	0.034	−0.069	
	Agreeableness		−1.197		−0.045	0.038	−0.031	
	Neuroticism		4.057 ***		0.141	0.035	0.111	
C. Dependent variable: Leisure living difficulties								
I.	Constant				1.716	0.076		
	Control variables	4.329 **		4, 1865				0.009
	Age		2.177 *		0.006	0.003	0.052	
	Gender		1.169		0.044	0.037	0.027	
	Duration of stay		−3.575 ***		−0.003	0.001	−0.092	
	Status		1.768		0.073	0.041	0.046	
II.	Internet use	25.168 ***		3, 1862				0.039
	Online benefits		0.515		0.012	0.023	0.014	
	Online habits		−1.950		−0.046	0.023	−0.054	
	Online facilitation		−6.680 ***		−0.171	0.026	−0.177	
III.	Personality	14.785 ***		5, 1857				0.036
	Openness		−1.002		−0.033	0.033	−0.024	
	Conscientiousness		0.690		0.023	0.034	0.018	
	Extraversion		−0.590		−0.019	0.033	−0.015	
	Agreeableness		−3.702 ***		−0.136	0.037	−0.096	
	Neuroticism		4.891 ***		0.165	0.034	0.135	
D. Dependent variable: Local viewpoints difficulties								
I.	Constant				2.547	0.092		
	Control variables	3.490 **		4, 1865				0.007
	Age		2.267 *		0.008	0.003	0.054	
	Gender		−1.858		−0.083	0.045	−0.043	
	Duration of stay		−2.824 **		−0.003	0.001	−0.073	
	Status		1.581		0.079	0.050	0.041	
II.	Internet use	9.703 ***		3, 1862				0.015
	Online benefits		−3.928 ***		−0.112	0.029	−0.108	
	Online habits		−0.025		−0.001	0.029	−0.001	
	Online facilitation		−1.150		−0.036	0.031	−0.031	
III.	Personality	12.264 ***		5, 1857				0.031
	Openness		−1.402		−0.057	0.041	−0.035	
	Conscientiousness		−2.752 **		−0.113	0.041	−0.073	
	Extraversion		−0.828		−0.033	0.040	−0.022	
	Agreeableness		0.050		0.002	0.045	0.001	
	Neuroticism		4.163 ***		0.171	0.041	0.117	
E. Dependent variable: Daily living difficulties								
I.	Constant				1.706	0.088		
	Control variables	8.104 ***		4, 1865				0.017
	Age		5.201 ***		0.017	0.003	0.123	
	Gender		−0.688		−0.029	0.043	−0.016	
	Duration of stay		−1.979 *		−0.002	0.001	−0.051	
	Status		3.041 **		0.144	0.047	0.078	
II.	Internet use	6.071 ***		3, 1862				0.010
	Online benefits		0.279		0.008	0.027	0.008	
	Online habits		−0.653		−0.018	0.027	−0.018	
	Online facilitation		−3.485 ***		−0.104	0.030	−0.094	
III.	Personality	24.903 ***		5, 1857				0.061
	Openness		1.118		0.043	0.038	0.027	
	Conscientiousness		3.193 ***		0.123	0.039	0.083	
	Extraversion		−1.158		−0.044	0.038	−0.030	
	Agreeableness		−7.848 ***		−0.330	0.042	−0.202	
	Neuroticism		4.452 ***		0.172	0.039	0.123	
F. Dependent variable: Responsive difficulties								
I.	Constant				2.337	0.088		
	Control variables	1.150		4, 1865				0.002
	Age		1.439		0.005	0.003	0.034	
	Gender		−0.208		−0.009	0.043	−0.005	
	Duration of stay		−1.449		−0.002	0.001	−0.038	
	Status		1.633		0.078	0.048	0.042	
II.	Internet use	9.475 ***		3, 1862				0.015
	Online benefits		0.617		0.017	0.027	0.017	
	Online habits		−1.984 *		−0.054	0.027	−0.056	
	Online facilitation		−3.652 ***		−0.109	0.030	−0.098	
III.	Personality	22.949 ***		5, 1857				0.057
	Openness		0.897		0.034	0.038	0.022	
	Conscientiousness		−0.413		−0.016	0.039	−0.011	
	Extraversion		−3.248 ***		−0.123	0.038	−0.084	
	Agreeableness		−4.391 ***		−0.186	0.042	−0.114	
	Neuroticism		4.740 ***		0.184	0.039	0.132	
G. Dependent variable: Suppressive difficulties								
I.	Constant				1.559	0.075		
	Control variables	6.797 ***		4, 1865				0.014
	Age		4.681 ***		0.013	0.003	0.111	
	Gender		1.536		0.057	0.037	0.036	
	Duration of stay		−0.362		0.000	0.001	−0.009	
	Status		0.773		0.031	0.041	0.020	
II.	Internet use	15.231 ***		3, 1862				0.024
	Online benefits		0.976		0.023	0.023	0.027	
	Online habits		−2.001 *		−0.047	0.023	−0.055	
	Online facilitation		−5.109 ***		−0.130	0.025	−0.136	
III.	Personality	7.402 ***		5, 1857				0.019
	Openness		−0.077		−0.003	0.033	−0.002	
	Conscientiousness		0.217		0.007	0.034	0.006	
	Extraversion		1.080		0.036	0.033	0.028	
	Agreeableness		−3.697 ***		−0.136	0.037	−0.097	
	Neuroticism		3.056 **		0.103	0.034	0.086	

Notes. *n* = 1870, *t* = for within-set predictors, df = degrees of freedom, B = unstandardized coefficients, SE = standard error, and β = standardized coefficients. Age is in years. Gender: 0 = female, 1 = male. Duration of stay is in months. Status: 0 = short-term exchange, 1 = degree-seeking. * *p* < 0.05, ** *p* < 0.01, *** *p* < 0.001.

**Table 4:** Moderation analysis and simple slopes models of study abroad difficulties, internet use, and openness.

Full Regression Model	β	SE	*t*	*p*	LLCI	ULCI
Predictor variables						
Constant	0.013	0.023	0.58	0.563	−0.032	0.059
Covariates						
Age	0.074	0.023	3.15	0.002	0.028	0.120
Gender	−0.006	0.023	−0.27	0.789	−0.051	0.039
Duration of stay	−0.074	0.025	−2.90	0.004	−0.123	−0.024
Status	0.081	0.025	3.21	0.001	0.032	0.131
Main effects						
Internet use	−0.156	0.024	−6.63	<0.001	−0.202	−0.110
Openness	−0.078	0.023	−3.33	0.001	−0.123	−0.032
Two-way interaction						
Internet use X Openness	−0.058	0.022	−2.65	0.008	−0.101	−0.015
Model fit	**R^2^**	**Adjusted R^2^**	**f^2^**			
	0.053	0.050	0.06			
**Simple slopes models**	**β**	**SE**	***t***	***p***		
Groupings						
+2 SD (*n* = 27)						
Intercept	−0.142					
Slope	−0.272	0.055	−4.94	<0.001	−0.380	−0.164
Mean (*n* = 1807)						
Intercept	0.013					
Slope	−0.156	0.024	−6.63	<0.001	−0.202	−0.110
−2 SD (*n* = 36)						
Intercept	0.168					
Slope	−0.040	0.050	−0.81	0.416	−0.138	0.057
Simple slopes difference (+2 SD, −2 SD)						
	−0.231	0.027	−8.63	<0.001		

Notes. All variables and predictors were standardized and centered prior to computing. *n* = 1870. β = standardized coefficients, SE = standard error, LLCI = lower level confidence interval, and ULCI = upper level confidence interval. Age is in years. Gender: 0 = female, 1 = male. Duration of stay is in months. Status: 0 = short-term exchange, 1 = degree-seeking.

**Table 5:** Moderation analysis and simple slopes models of study abroad difficulties, internet use, and conscientiousness.

Full Regression Model	β	SE	*t*	*p*	LLCI	ULCI
Predictor variables						
Constant	0.008	0.023	0.34	0.733	−0.037	0.053
Covariates						
Age	0.067	0.023	2.86	0.004	0.021	0.112
Gender	0.001	0.023	0.05	0.963	−0.044	0.046
Duration of stay	−0.075	0.025	−2.98	0.003	−0.125	−0.026
Status	0.088	0.025	3.51	<0.001	0.039	0.138
Main effects						
Internet use	−0.143	0.024	−6.06	<0.001	−0.189	−0.097
Conscientiousness	−0.143	0.023	−6.20	0.001	−0.188	−0.098
Two-way interaction						
Internet use X Conscientiousness	−0.040	0.022	−1.81	0.070	−0.188	0.003
Model fit	**R^2^**	**Adjusted R^2^**	**f^2^**			
	0.065	0.062	0.07			
**Simple slopes models**	**β**	**SE**	***t***	***p***		
Groupings						
+2 SD (*n* = 48)						
Intercept	−0.278					
Slope	−0.223	0.054	−4.10	<0.001	−0.330	−0.117
Mean (*n* = 1781)						
Intercept	0.008					
Slope	−0.143	0.024	−6.06	<0.001	−0.189	−0.097
−2 SD (*n* = 41)						
Intercept	0.294					
Slope	−0.062	0.050	−1.25	0.213	−0.159	0.036
Simple slopes difference (+2 SD, −2 SD)						
	−0.161	0.027	−6.04	<0.001		

Notes. All variables and predictors were standardized and centered prior to computing. *n* = 1870. β = standardized coefficients, SE = standard error, LLCI = lower level confidence interval, and ULCI = upper level confidence interval. Age is in years. Gender: 0 = female, 1 = male. Duration of stay is in months. Status: 0 = short-term exchange, 1 = degree-seeking.

**Table 6:** Moderation analysis and simple slopes models of study abroad difficulties, internet use, and extraversion.

Full Regression Model	β	SE	*t*	*p*	LLCI	ULCI
Predictor variables						
Constant	0.017	0.023	0.74	0.462	−0.028	0.062
Covariates						
Age	0.072	0.023	3.09	0.002	0.026	0.117
Gender	−0.008	0.023	−0.38	0.707	−0.053	0.036
Duration of stay	−0.073	0.025	−2.91	0.004	−0.122	−0.024
Status	0.075	0.025	3.01	0.003	0.026	0.124
Main effects						
Internet use	−0.137	0.023	−5.86	<0.001	−0.182	−0.091
Extraversion	−0.151	0.023	−6.56	<0.001	−0.196	−0.106
Two-way interaction						
Internet use X Extraversion	−0.073	0.021	−3.50	<0.001	−0.114	−0.032
Model fit	**R^2^**	**Adjusted R^2^**	**f^2^**			
	0.071	0.068	0.08			
**Simple slopes models**	**β**	SE	***t***	***p***		
Groupings						
+2 SD (*n* = 47)						
Intercept	−0.285					
Slope	−0.283	0.054	−5.20	<0.001	−0.390	−0.177
Mean (*n* = 1782)						
Intercept	0.017					
Slope	−0.137	0.023	−5.86	<0.001	−0.182	−0.091
−2 SD (*n* = 41)						
Intercept	0.318					
Slope	0.010	0.049	0.20	0.840	−0.087	0.106
Simple slopes difference (+2 SD, −2 SD)						
	−0.293	0.027	−11.02	<0.001		

Notes. All variables and predictors were standardized and centered prior to computing. *n* = 1870. β = standardized coefficients, SE = standard error, LLCI = lower level confidence interval, and ULCI = upper level confidence interval. Age is in years. Gender: 0 = female, 1 = male. Duration of stay is in months. Status: 0 = short-term exchange, 1 = degree-seeking.

**Table 7:** Moderation analysis and simple slopes models of study abroad difficulties, internet use, and agreeableness.

Full Regression Model	β	SE	*t*	*p*	LLCI	ULCI
Predictor variables						
Constant	0.013	0.023	0.59	0.556	−0.031	0.058
Covariates						
Age	0.068	0.023	2.97	0.003	0.023	0.113
Gender	−0.011	0.022	−0.50	0.617	−0.055	0.033
Duration of stay	−0.074	0.025	−3.00	0.003	−0.123	−0.026
Status	0.094	0.025	3.80	<0.001	0.046	0.143
Main effects						
Internet use	−0.125	0.023	−5.42	<0.001	−0.170	−0.080
Agreeableness	−0.216	0.023	−9.47	<0.001	−0.261	−0.171
Two-way interaction						
Internet use X Agreeableness	−0.058	0.021	−2.75	0.006	−0.100	−0.017
Model fit	**R^2^**	**Adjusted R^2^**	**f^2^**			
	0.091	0.087	0.10			
**Simple slopes models**	**β**	**SE**	***t***	***p***		
Groupings						
+2 SD (*n* = 39)						
Intercept	−0.419					
Slope	−0.242	0.054	−4.50	<0.001	−0.347	−0.136
Mean (*n* = 1789)						
Intercept	0.013					
Slope	−0.125	0.023	−5.42	<0.001	−0.170	−0.080
−2 SD (*n* = 42)						
Intercept	0.445					
Slope	−0.008	0.049	−0.16	0.872	−0.104	0.088
Simple slopes difference (+2 SD, −2 SD)						
	−0.234	0.026	−8.92	<0.001		

Notes. All variables and predictors were standardized and centered prior to computing. *n* = 1870. β = standardized coefficients, SE = standard error, LLCI = lower level confidence interval, and ULCI = upper level confidence interval. Age is in years. Gender: 0 = female, 1 = male. Duration of stay is in months. Status: 0 = short-term exchange, 1 = degree-seeking.

**Table 8:** Moderation analysis and simple slopes models of study abroad difficulties, internet use, and neuroticism.

Full Regression Model	β	SE	*t*	*p*	LLCI	ULCI
Predictor variables						
Constant	0.011	0.022	0.50	0.618	−0.033	0.055
Covariates						
Age	0.072	0.023	3.18	0.001	0.028	0.117
Gender	0.006	0.022	0.27	0.787	−0.038	0.050
Duration of stay	−0.069	0.025	−2.81	0.005	−0.117	−0.021
Status	0.097	0.025	3.93	<0.001	0.048	0.145
Main effects						
Internet use	−0.123	0.023	−5.37	<0.001	−0.167	−0.078
Neuroticism	0.242	0.023	10.69	<0.001	0.197	0.286
Two-way interaction						
Internet use X Neuroticism	0.060	0.022	2.71	0.007	0.017	0.103
Model fit	**R^2^**	**Adjusted R^2^**	**f^2^**			
	0.106	0.103	0.12			
**Simple slopes models**	**β**	**SE**	***t***	***p***		
Groupings						
+2 SD (*n* = 24)						
Intercept	0.495					
Slope	−0.003	0.053	−0.06	0.955	−0.108	0.101
Mean (*n* = 1790)						
Intercept	0.011					
Slope	−0.123	0.023	−5.37	<0.001	−0.167	−0.078
−2 SD (*n* = 56)						
Intercept	−0.473					
Slope	−0.242	0.048	−5.02	<0.001	−0.337	−0.147
Simple slopes difference (+2 SD, −2 SD)						
	0.239	0.026	9.37	<0.001		

Notes. All variables and predictors were standardized and centered prior to computing. *n* = 1870. β = standardized coefficients, SE = standard error, LLCI = lower level confidence interval, and ULCI = upper level confidence interval. Age is in years. Gender: 0 = female, 1 = male. Duration of stay is in months. Status: 0 = short-term exchange, 1 = degree-seeking.

**Table 9:** Summary of hierarchical multiple regressions results.

Stage	Variables	Overall	Academic	Leisure	Viewpoints	Daily	Responsive	Suppressive
1	Age	✓(+)		✓(+)	✓(+)	✓(+)		✓(+)
	Gender							
	Duration of stay	✓(−)		✓(−)	✓(−)	✓(−)		
	Status	✓(+)	✓(+)			✓(+)		
2	Online benefits				✓(−)			
	Online habits						✓(−)	✓(−)
	Online facilitation	✓(−)	✓(−)	✓(−)		✓(−)	✓(−)	✓(−)
3	Openness							
	Conscientiousness		✓(−)		✓(−)	✓(+)		
	Extraversion		✓(−)				✓(−)	
	Agreeableness	✓(−)		✓(−)		✓(−)	✓(−)	✓(−)
	Neuroticism	✓(+)	✓(+)	✓(+)	✓(+)	✓(+)	✓(+)	✓(+)

Notes. ✓ = significant predictors. (−) negative or (+) positive association with the dependent variable.

## Data Availability

Data for the current study is available at https://doi.org/10.6084/m9.figshare.14812068.v1 (accessed on 20 June 2021).
